# Ultrasound-Guided Injection of a Corticosteroid Technique for the Treatment of Degenerative Meniscal Tear

**DOI:** 10.1016/j.eats.2024.103231

**Published:** 2024-09-24

**Authors:** François Duprat, Dany Mouarbes, Emilie Berard, Pierre Thomas, Johan Laffort, Etienne Cavaignac, Marie Faruch Bilfeld

**Affiliations:** a Department of Orthopedic Surgery and Trauma, Pierre-Paul Riquet Hospital, Toulouse, France; b Department of Epidemiology, Health Economics and Public Health, UMR 1027 INSERM–University of Toulouse III, Toulouse University Hospital (CHU), Toulouse, France; c Physical Medicine and Rehabilitation, Pierre-Paul Riquet Hospital, Toulouse, France; d Department of Radiology, CHU de Toulouse, Toulouse, France

## Abstract

Degenerative meniscal tear is a chronic disorder that presents with knee pain, swelling, and loss of motion. It usually develops slowly on meniscal tissue that already has macroscopic and ultra-structural changes that affect its resistance to load. Conservative management, such as corticosteroid infiltration, is currently advocated as a first-line approach. However, it has been empirically observed that intra-articular injections do not appropriately alleviate pain because they do not target the trigger area of the meniscus and are quickly cleared from joints via synovial capillaries and lymphatic drainage. In recent years, there has been increased interest in the use of ultrasound guidance for meniscal and perimeniscal injection. Cadaveric specimens have offered optimal visualization of local anatomic structures, permitting safe and precise percutaneous delivery of medication in the meniscus. The vascularization is located mainly in the peripheral third of the meniscus, particularly on the perimeniscal capillary plexus. This is why this area has healing potential, in contrast to the free zone of the meniscus. The ultrasound-guided infiltration of the meniscal wall is a technique that is more targeted and more effective on the trigger zone.

Degenerative meniscal tear is a chronic disorder that presents with knee pain, swelling, and loss of motion (Figs 1 and 2).^1^ It usually develops slowly on meniscal tissue that already has macroscopic and ultrastructural changes that affect its resistance to load.^2^ There is growing evidence that knee arthroscopy may not be efficacious in the treatment of degenerative meniscal tear, and its utilization has decreased alongside reimbursement rates in the United States.^3^ Conservative management, such as corticosteroid infiltration, is currently advocated as a first-line approach.^4^^,^^5^ However, it has been empirically observed that intra-articular injections do not appropriately alleviate pain because they do not target the trigger area of the meniscus and are quickly cleared from joints via synovial capillaries and lymphatic drainage.^6^^,^^7^ In the recent years, there has been increased interest in the use of ultrasound guidance for meniscal and perimeniscal injection.^8^, ^9^, ^10^ Cadaveric specimens have offered optimal visualization of local anatomic structures, permitting safe and precise percutaneous delivery of medication in the meniscus.^2^^,^^8^^,^^11^ The vascularization is located mainly in the peripheral third of the meniscus, particularly on the perimeniscal capillary plexus (Fig 1).^12^^,^^13^ This is why this area has healing potential, in contrast to the free zone of the meniscus.^12^ Because the menisci innervation follows the blood supply, nerve fibers are found primarily in the peripheral vascular zone covering the outer third of the meniscus, whereas the inner two-thirds of the menisci contain no nerves.^14^ Owing to the peripheral location of the free nerve endings acting as nociceptors in meniscal pain, a meniscus-targeted injection with corticosteroid treats the “trigger” tissue rather than the whole joint.^14^ With its anti-inflammatory, fibrotic, and analgesic effects, it can provide a timely and lasting clinical improvement by interrupting the inflammatory cascade and reducing vascular permeability at the peripheral border of the menisci.^14^^,^^15^ The injection is more targeted and then more effective.^16^

**Fig 1:**
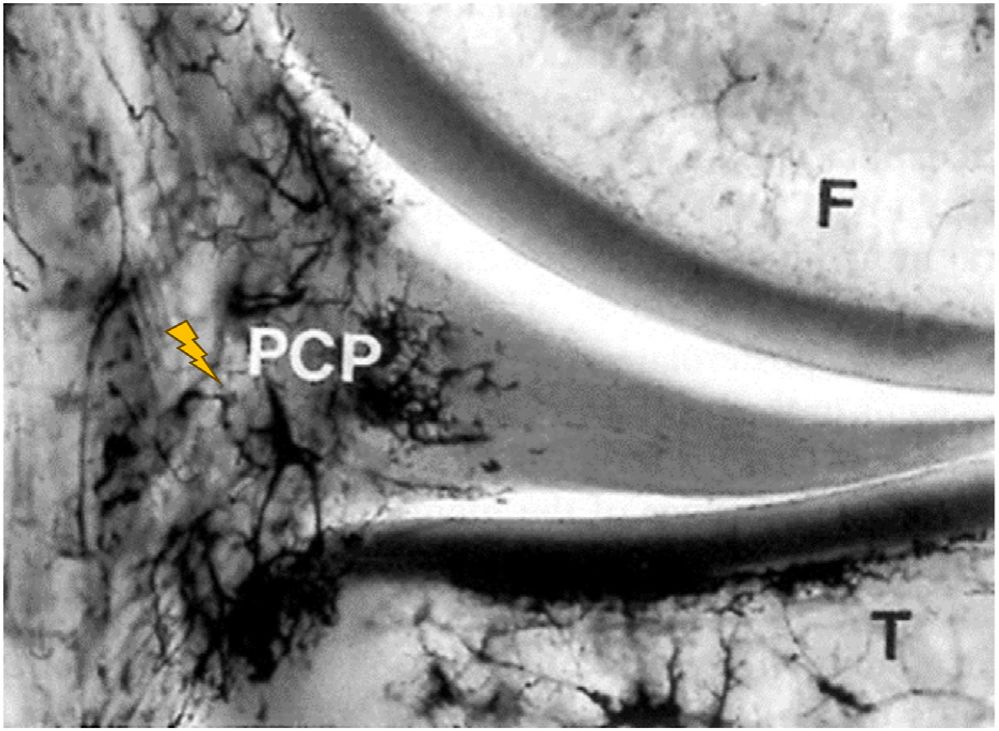
Vascularization of the meniscus (coronal view). The ultrasound-guided injection is targeted on the trigger area (yellow flash), which is located near the perimeniscal capillary plexus. Reprinted from Arnoczky and Warren^15^ by permission of Sage Publications. (F, femur; PCP, perimeniscal capillary plexus; T, tibia.)

**Fig 2:**
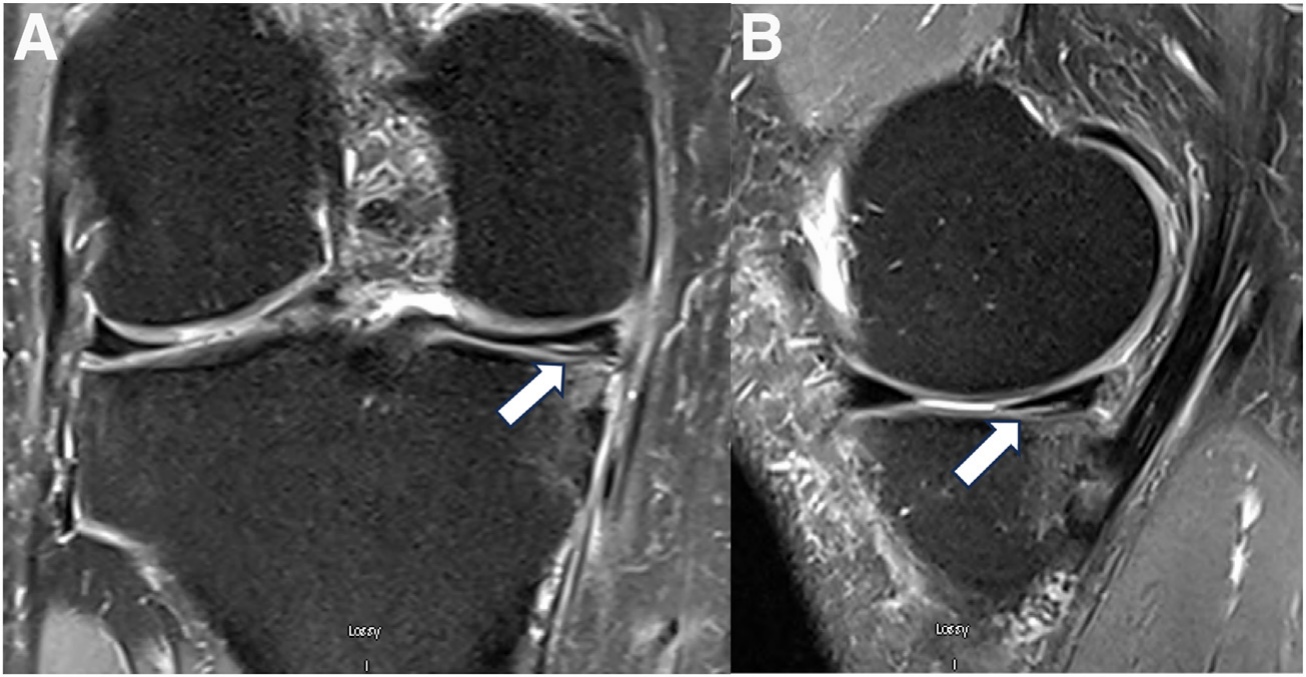
Degenerative grade 2 medial meniscal tear of the right knee in a 57-year-old man. The tear (white arrow) is located in the posterior horn of the medial meniscus on the coronal view (A) and sagittal view (B) of magnetic resonance imaging of the knee.

## Infiltration Technique

Our case is a 57-year-old man with a grade 2 degenerative medial meniscal tear of the right knee located in the posterior horn of the medial meniscus. Magnetic resonance imaging is presented in Figure 2.

### Infiltration Preparation

**Fig 3:**
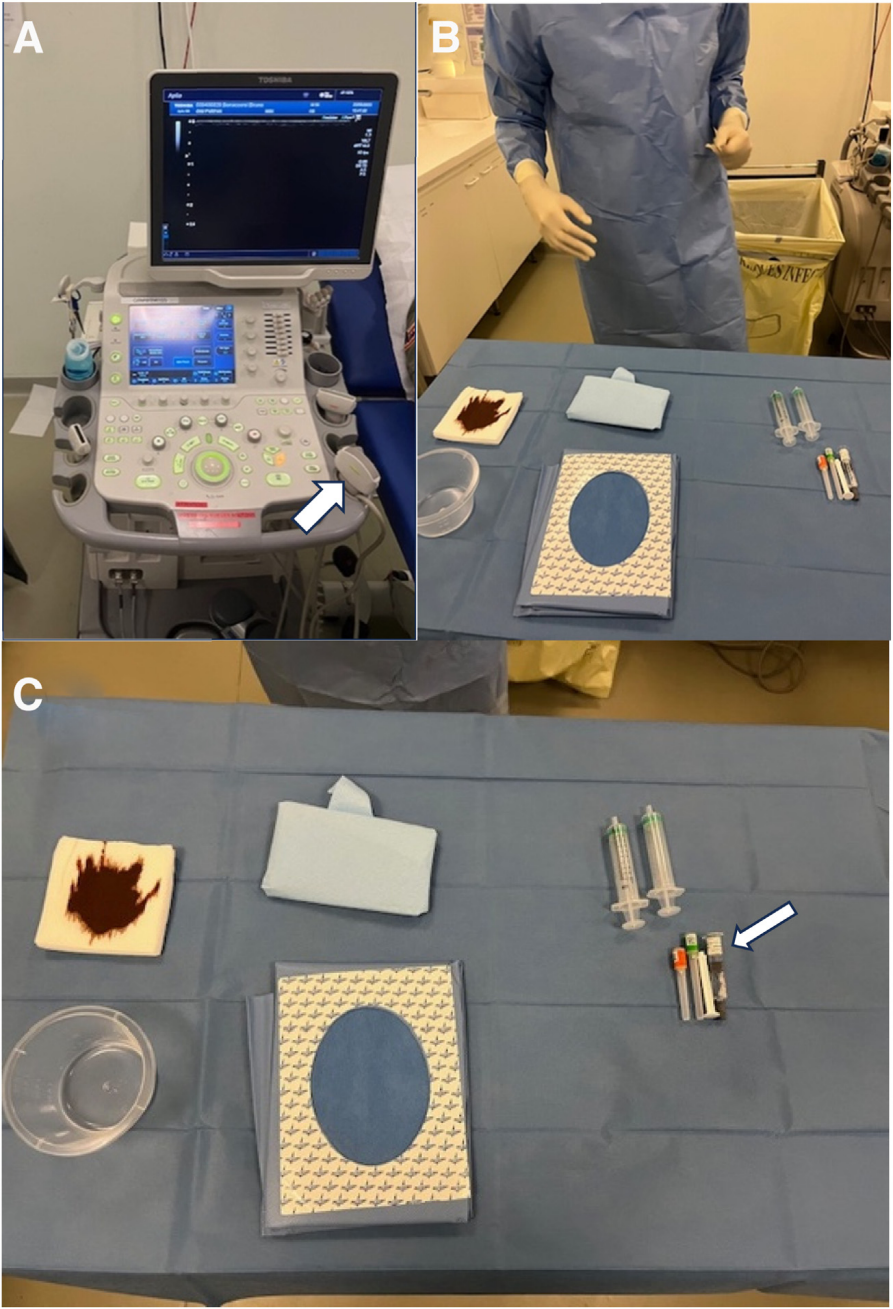
Equipment necessary for infiltration. (A) Toshiba ultrasound with a linear 18-MHz probe (white arrow). (B) Installation of the table under aseptic conditions. (C) View of the table: note that the material is very simple. We use 1 mL betamethasone and a 21-gauge needle for the infiltration (white arrow).

Infiltrations are carried out by a radiologist trained in osteoarticular pathology. We use an ultrasound (Toshiba) using a linear 18-MHz probe under strict aseptic conditions (Fig 3 A and B). The equipment necessary for infiltration is very simple (Fig 3C): on a sterile table, we take a sterile holed field, compresses and antiseptic (betadine), 2 mL of local anesthesia (lidocaine), 1 mL of corticosteroid (betamethasone), and a 21- and 25-gauge needle.

**Fig 4:**
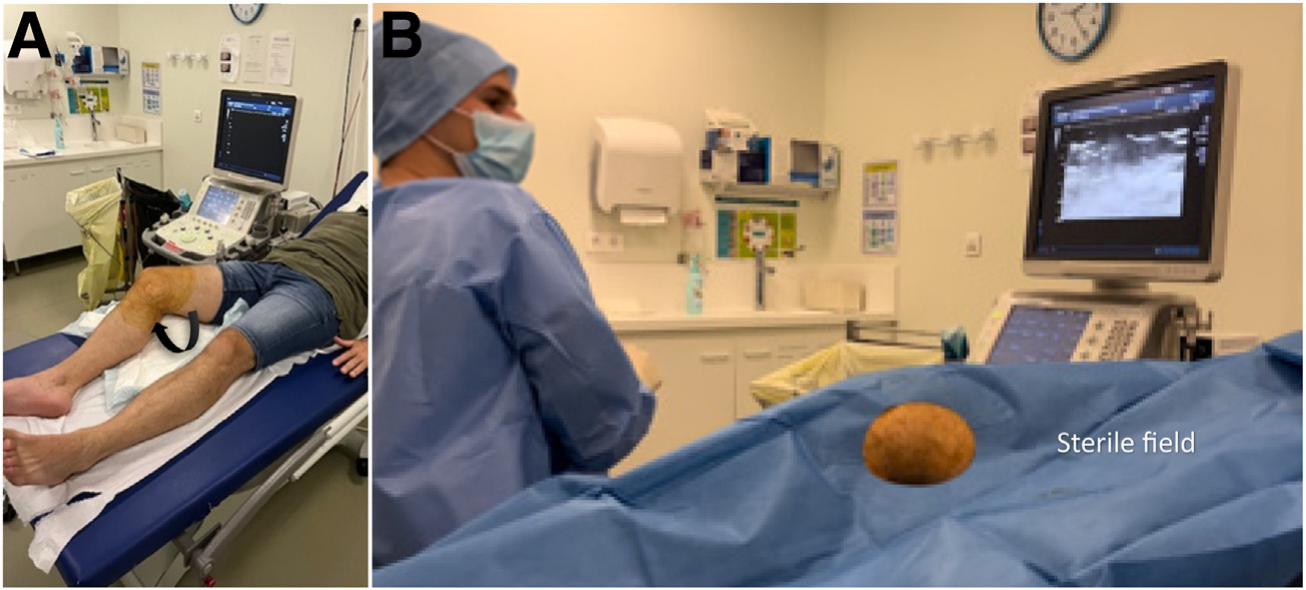
Installation in supine position. (A) The right knee is flexed at 30° and in the valgus position (black arrow) to see the joint better. (B) Knee coverage with a sterile field.

To optimize sonographic visualization and meniscal access, the knee is flexed to 30° and a gravity-induced valgus stress is imparted on the joint (Fig 4A).

After disinfection in 4 stages, the knee is covered by a holed field, and the procedure of infiltration can begin (Fig 4B).

### Echo Infiltration of the Meniscal Wall

**Fig 5:**
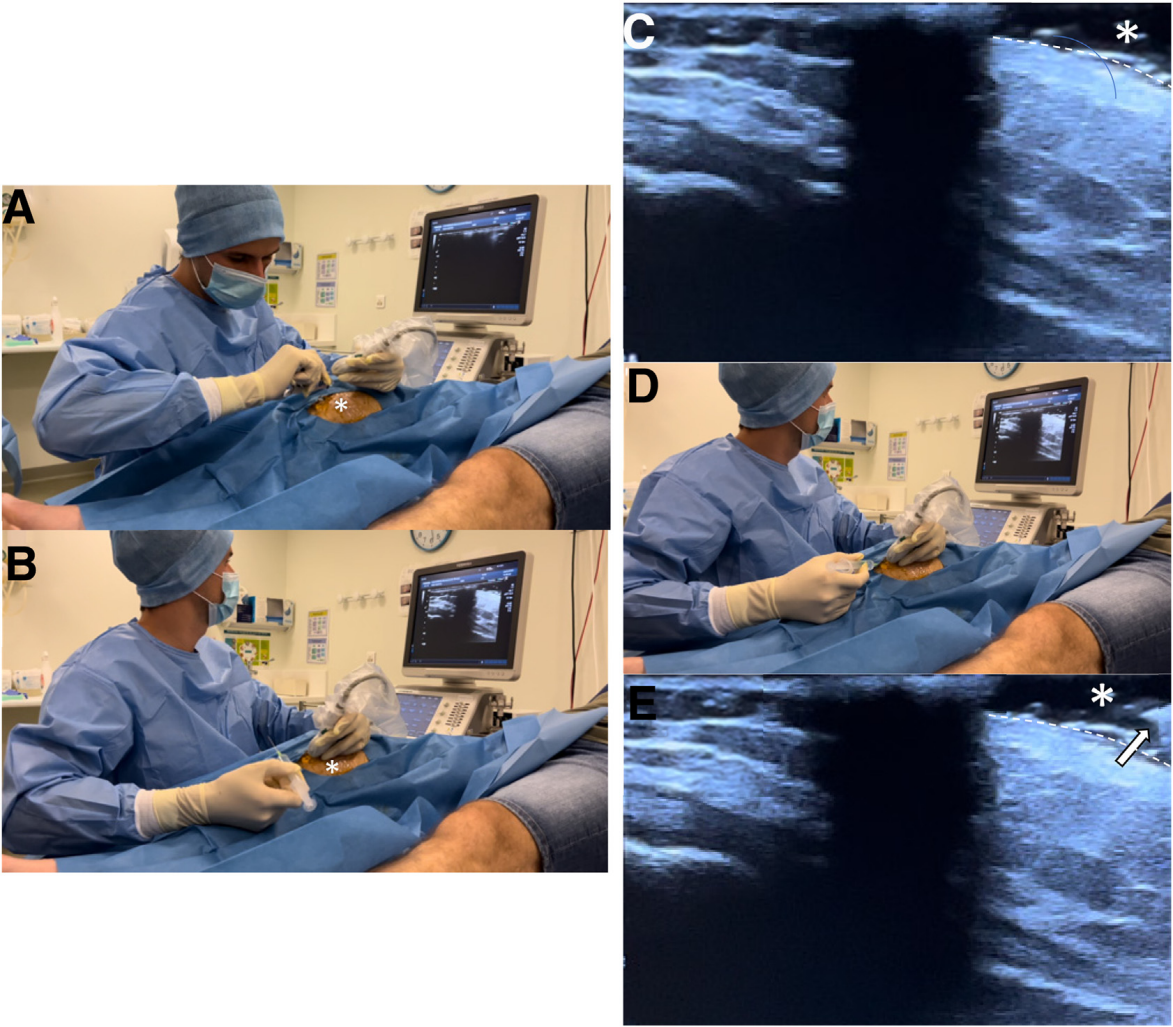
Ultrasound check and local anesthesia for a medial meniscal tear of the right knee (longitudinal view). (A, B) The technique used by our team consists of interposing a “gel pad” (∗) between the probe and the skin, which allows locating the 21-gauge needle and giving it a perfect orientation before local anesthesia with 2 mL lidocaine, thus making the procedure not painful. (C) The “gel pad” (∗) can be seen in the right corner, which is a hypoechogenic area above the skin (white dotted line). (D) Then we easily find the needle in the gel pad. (E) The needle (white arrow) in the “gel pad” (∗) can be seen in the simultaneous ultrasound imaging.

**Fig 6:**
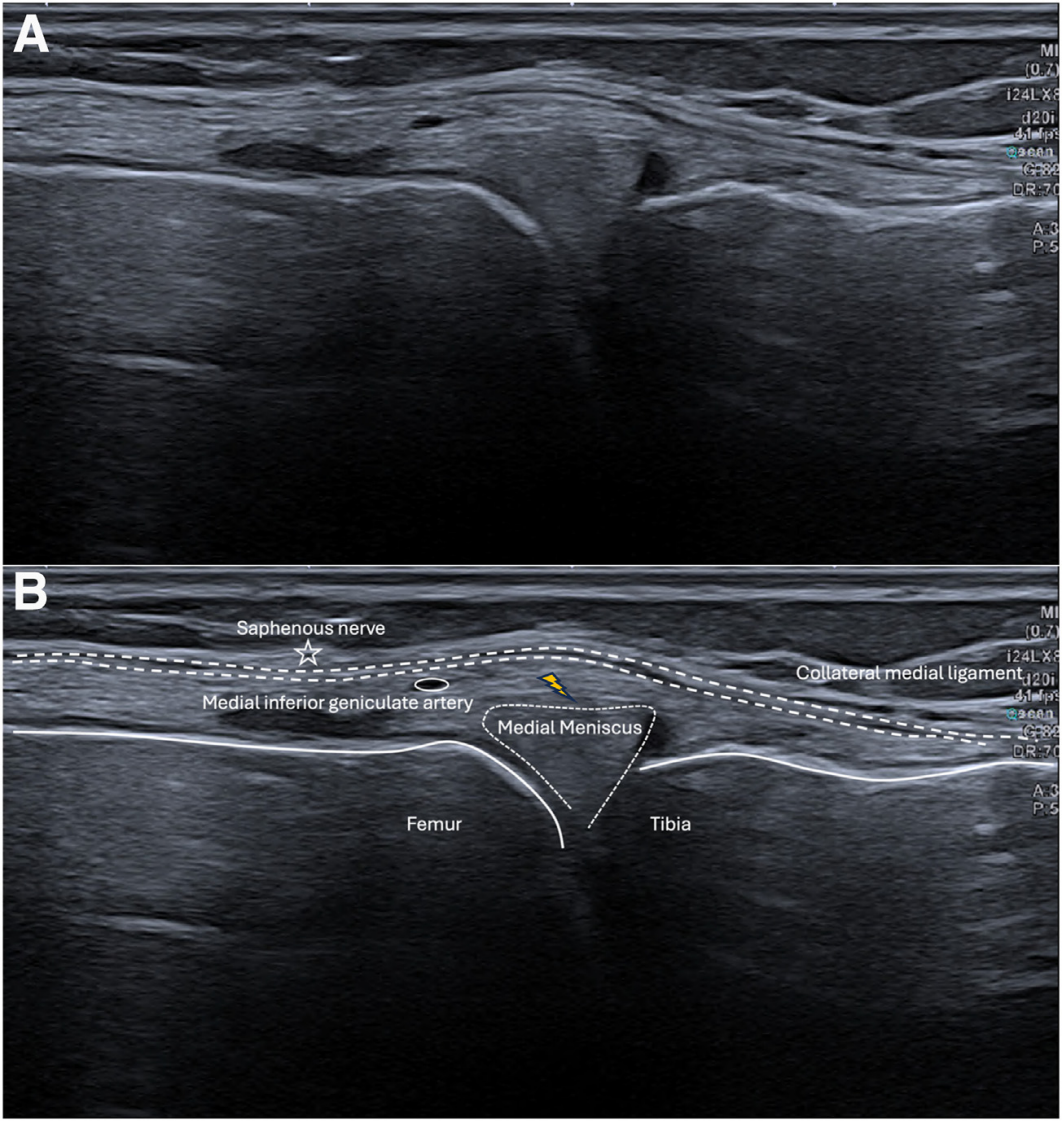
Different structures to spot during infiltration for a medial meniscal tear of the right knee in longitudinal view (A, B). The anterior aspect of the medial collateral ligament is used as anatomic landmarks for transducer placement during injections. It is important to identify the saphenous nerve and the medial inferior geniculate artery before infiltration to avoid them. The meniscal wall (yellow flash) is located between the articular capsule (double dotted line) and the periphery of the medial meniscus.

Initial ultrasound (US) examination of the medial menisci is performed by placing the transducer both longitudinally and transversely to obtain visualization of the tibiofemoral joint space and body of the meniscus (Fig 5). Thereafter, the regional saphenous nerve is identified sonographically and its location is marked. Doppler US is routinely used prior to injection to identify the medial inferior geniculate artery (Fig 6).

**Fig 7:**
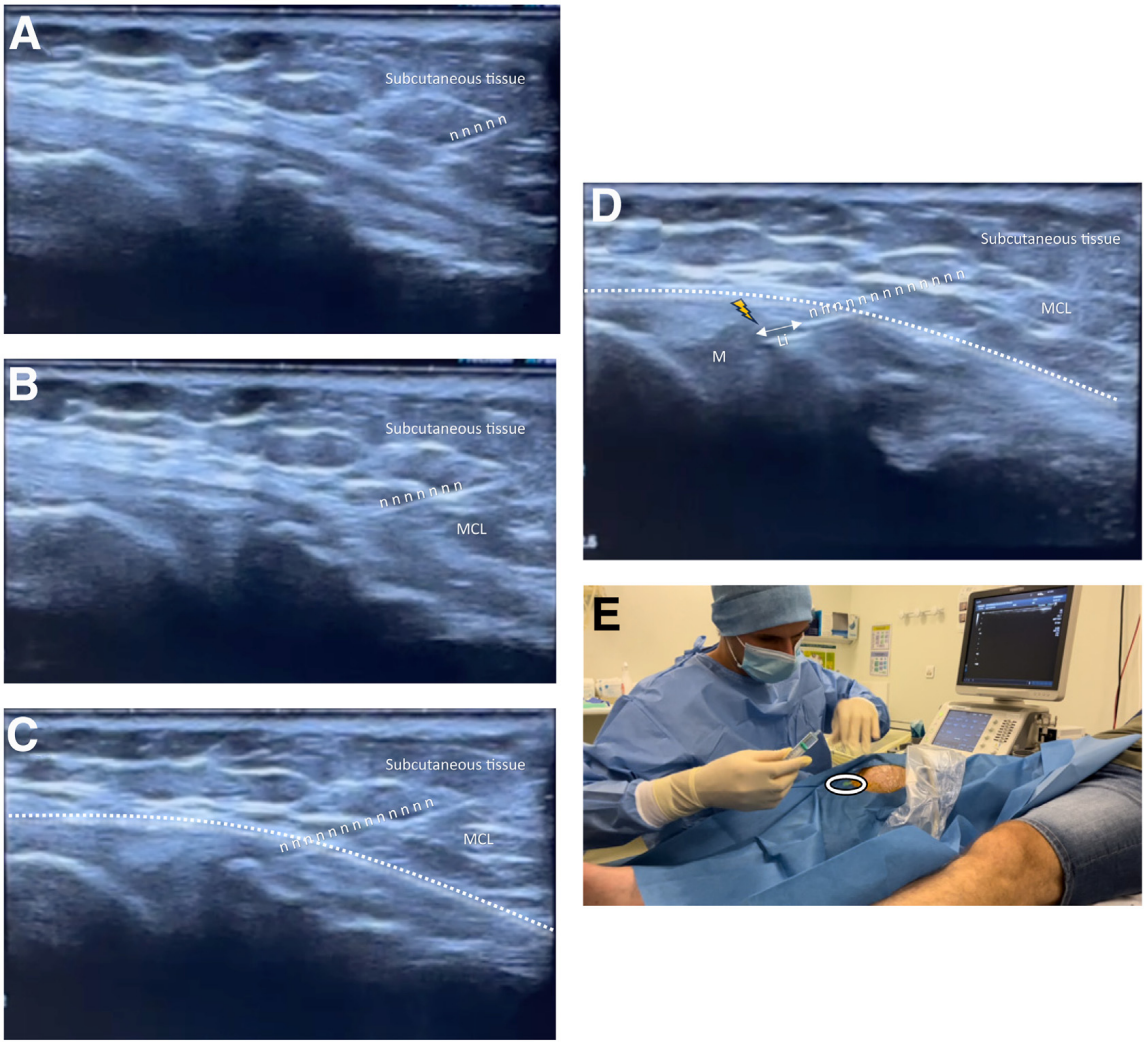
Local anesthesia is performed with 2 mL lidocaine for a medial meniscal tear of the right knee. By a plan-by-plan approach in a longitudinal view, from cranial to caudal, the needle is progressed from the gel pad through the skin and subcutaneous tissue (A), the medial collateral ligament (B), and the articular capsule (dotted line, C) until reaching the target area of “the meniscal wall” (yellow flash, D). The injection of lidocaine (Li) can be seen in contact with the meniscal wall (D). Once the target zone is reached, the syringe is then withdrawn without removing the needle (white circle, E).

**Fig 8:**
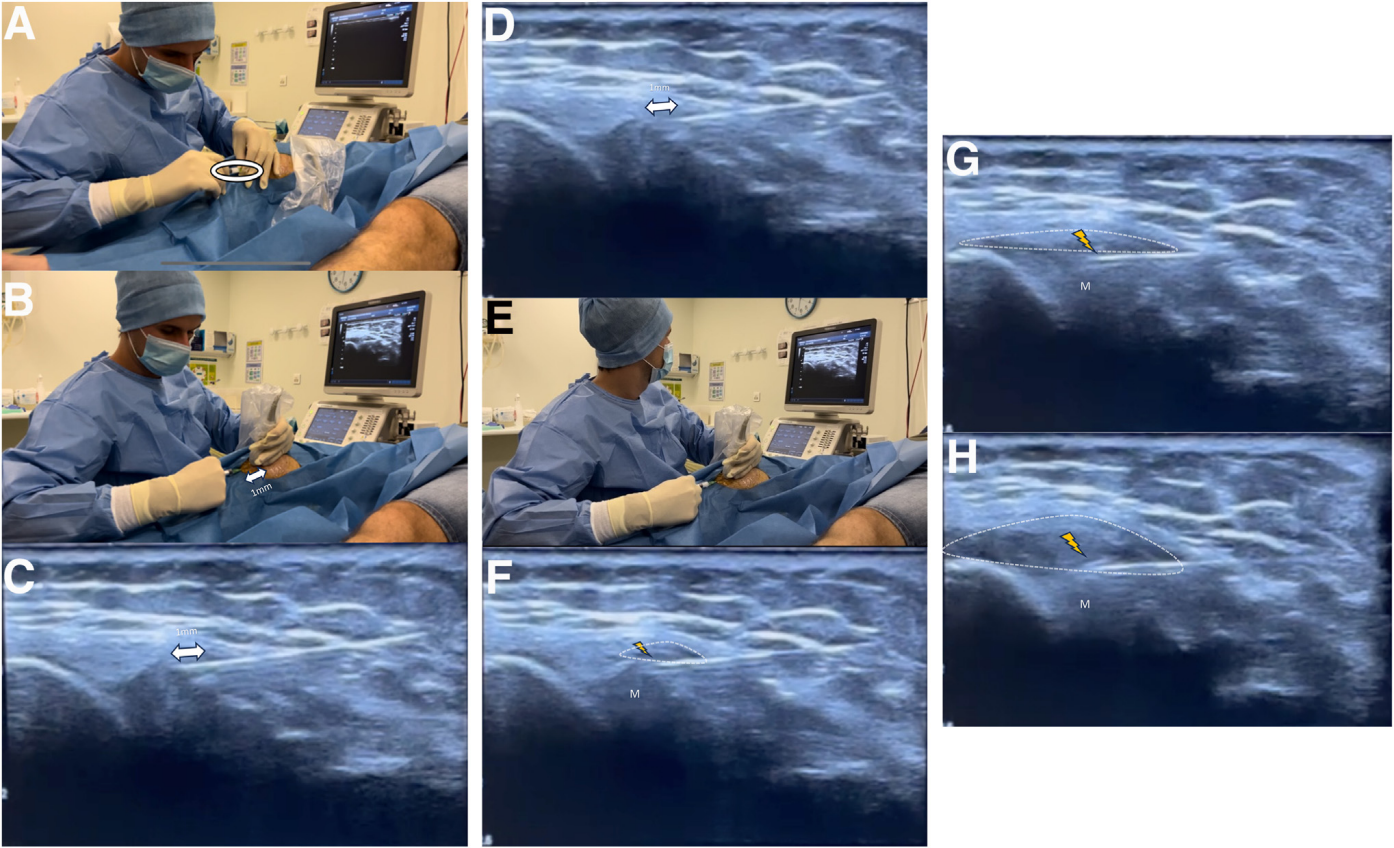
Betamethasone infiltration for a medial meniscal tear of the right knee (longitudinal view). (A) Placement of the syringe of betamethasone (white circle) without moving the needle. (B) Under ultrasound control, the needle is slightly retracted by 1 mm before infiltration. (C) Simultaneous ultrasound image with the needle in contact with the meniscus and then retracted by 1 mm before infiltration (D). (E) We proceed with the infiltration of 1 mL betamethasone in the meniscal wall. You can see the hypoechogenic product diffusing progressively (F-H) between the articular capsule and the periphery of the meniscus. (M, meniscus.)

**Fig 9:**
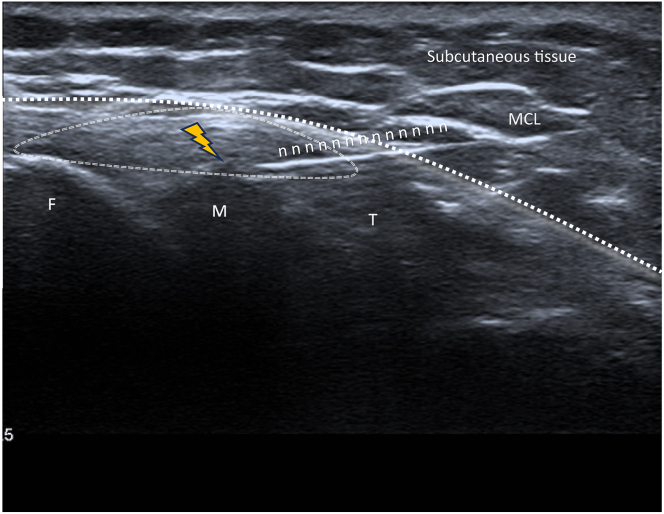
Final infiltration image for a medial meniscal tear of the right knee (longitudinal view). You can see the meniscus (M) between the femur (F) and the tibia (T), the path of the needle (n) through subcutaneous tissue, the medial collateral ligament (MCL), and articular capsule (dotted line) until the trigger area of “the meniscal wall” (yellow flash). The injection is very safe, targeted, and therefore more effective.

After identification of the regional at-risk structures, the US-guided meniscal wall injection is performed. The anterior aspect of the medial collateral ligament is used as anatomic landmarks for transducer placement during injections (Fig 6). The first step consists of performing local anesthesia with 2 mL lidocaine injected into the subcutaneous tissue and close to the wall of the meniscus using a 25-gauge needle. Then, using an in-plane approach, a 21-gauge needle is advanced under sonographic guidance from cranial to caudal into the medial meniscus wall (Fig 7). Once the needle touches the meniscus wall, it is slightly retracted by 1 mm, and an injection of 1 mL betamethasone in the meniscal wall tissue is performed (Fig 8). The injectate administration is confirmed by real-time visualization of bright echoes filling the perimeniscal area interposed between the meniscus wall and joint capsule (Fig 8, Fig 9, Video 1). The injection is directly followed by a simple 4-week rehabilitation program with 3 sessions per week performed and supervised by the physiotherapist: we begin with massage, cryotherapy, and exercise to improve passive and active knee mobility, and we progressively increase quadriceps strength and tonicity.

## Discussion

This technique was originally described by Bouvard and Juret,^17^ who showed that this infiltration was an effective treatment in 214 patients.

In recent years, there has been increased interest in the use of US guidance for therapeutic injections in the musculoskeletal system, with the advantages of real-time imaging, absence of radiation exposure, and convenience of performing the procedure portably.^8^ In particular, at the knee, US offers high spatial resolution and exquisite soft tissue delineation, making it an ideal guidance modality for both intra- and extra-articular injections.^9^^,^^10^ Three articles on cadaveric specimens validated the feasibility and safety of intrameniscal and perimeniscal injection under US guidance.^2^^,^^8^^,^^11^ Using cadaveric specimens, they demonstrate that US guidance, by offering optimal visualization of local anatomic structures, permits a safe and precise percutaneous delivery of medication in intrameniscal and perimeniscal tissue.

Moreover, the great advantage of this technique is targeting the trigger area of the meniscus with a small quantity of corticosteroids (1 mL), without dilution of the products in the synovial fluid and without the possible harmful effects that repeated intra-articular injections can cause. This technique provides excellent safety for the procedure, which is controlled under ultrasound (Table 1).

**Table 1:** Advantages/Disadvantages

Advantages	Disadvantages
Targets the trigger area	Requires skill level and training
Uses a small quantity of corticosteroids	Longer procedure than intra-articular infiltration
No dilution of the product in synovial fluid	Less accessible
Excellent safety of the procedure	

The disadvantage of this technique is that it requires training because the procedure is very precise and targeted, and it is longer than an intra-articular infiltration and possibly has a longer accessibility time (Table 1).

This technique requires some skills to avoid risky structures, such as the regional saphenous nerve and medial inferior genicular artery. Moreover, it is important to target a specific area that is between the meniscal wall and the articular capsule to avoid extracapsular injection (ineffective injection) or intrameniscal injection, which can lead to meniscus injury. There are some easy pearls to proceed to a safe, targeted, and effective infiltration (Table 2).

**Table 2:** Pearls and Pitfalls

Pitfalls	Pearls
Lesion of regional saphenous nerve is possible	Identify its path on ultrasound and mark before infiltration
Lesion of medial inferior genicular artery is possible	Identify this artery with the help of doppler before infiltration
Improper placement of ultrasound probe	Use the anterior aspect of the medial collateral ligament as anatomic landmarks for transducer placement during injections
Extracapsular injection	Touch the meniscal wall with the needle and slowly retract the needle by 1 mm Visualization of bright echo filling between the meniscal wall and capsule

## Disclosures

The authors declare the following financial interests/personal relationships which may be considered as potential competing interests: E.T. is a paid consultant with Arthrex and Amplitude, which have no link to the results of this study. All other authors (F.D., D.M., E.M., P.T., J.L., M.F.B.) declare that they have no known competing financial interests or personal relationships that could have appeared to influence the work reported in this paper.

## Supplementary Data

Video 1 This is a technique video for ultrasound-guided meniscal injection of corticosteroid (medial meniscal tear of the right knee). The patient is positioned in a supine position in the ultrasound room. We start with an ultrasound control in longitudinal view with the knee flexed at 30° and in the valgus position. The probe is placed perpendicularly to the medial femorotibial joint. We visualize the meniscus, which is a triangular hypoechogenic structure located between the tibia and the femur and its meniscal wall. It is necessary to identify the saphenous nerve and medial inferior geniculate artery to avoid them. Local anesthesia is performed with 2 mL lidocaine. Using the “gel pad” technique, we give the right orientation of the 21-gauge needle from cranial to caudal before crossing the skin plane, and anesthesia is performed plan by plan, starting with the skin and then the subcutaneous tissues, the medial collateral ligament, the articular capsule, and finally the target area: the meniscal wall. When the needle touches the periphery of the meniscus, the syringe is then withdrawn without removing the needle. Finally, the needle is slightly retracted by 1 mm, and we proceed with the injection of 1 mL betamethasone in the meniscal wall. Always under ultrasound control, we observe in real time the injection of corticosteroids. We see here the hypoechogenic product diffusing between the meniscal wall and the articular capsule. The injection is perfectly targeted and therefore more effective.
